# Untreated depression and tuberculosis treatment outcomes, quality of life and disability, Ethiopia

**DOI:** 10.2471/BLT.17.192658

**Published:** 2018-02-05

**Authors:** Fentie Ambaw, Rosie Mayston, Charlotte Hanlon, Girmay Medhin, Atalay Alem

**Affiliations:** a School of Public Health, College of Medicine and Health Sciences, Bahir Dar University, PO Box 1985, Bahir Dar, Ethiopia.; b Centre for Global Mental Health, King’s College London, London, England.; c Department of Psychiatry, Addis Ababa University, Addis Ababa, Ethiopia.

## Abstract

**Objective:**

To investigate the association between comorbid depression and tuberculosis treatment outcomes, quality of life and disability in Ethiopia.

**Methods:**

The study involved 648 consecutive adults treated for tuberculosis at 14 primary health-care facilities. All were assessed at treatment initiation (i.e. baseline) and after 2 and 6 months. We defined probable depression as a score of 10 or above on the nine-item Patient Health Questionnaire. Data on treatment default, failure and success and on death were obtained from tuberculosis registers. Quality of life was assessed using a visual analogue scale and we calculated disability scores using the World Health Organization’s Disability Assessment Scale. Using multivariate Poisson regression analysis, we estimated the association between probable depression at baseline and treatment outcomes and death.

**Results:**

Untreated depression at baseline was independently associated with tuberculosis treatment default (adjusted risk ratio, aRR: 9.09; 95% confidence interval, CI: 6.72 to 12.30), death (aRR: 2.99; 95% CI: 1.54 to 5.78), greater disability (*β*: 0.83; 95% CI: 0.67 to 0.99) and poorer quality of life (*β*: −0.07; 95% CI: −0.07 to −0.06) at 6 months. Participants with probable depression had a lower mean quality-of-life score than those without (5.0 versus 6.0, respectively; *P* < 0.001) and a higher median disability score (22.0 versus 14.0, respectively; *P* < 0.001) at 6 months.

**Conclusion:**

Untreated depression in people with tuberculosis was associated with worse treatment outcomes, poorer quality of life and greater disability. Health workers should be given the support needed to provide depression care for people with tuberculosis.

## Résumé

**Objectif:**

Étudier l'association entre la dépression et les résultats du traitement contre la tuberculose, la qualité de vie et le handicap en Éthiopie.

**Méthodes:**

L'étude a porté sur 648 adultes consécutifs traités contre la tuberculose dans 14 établissements de soins primaires. Tous ont été examinés au début du traitement (période de référence) puis au bout de 2 et 6 mois. Nous avons défini la dépression probable comme l'obtention d'un score de 10 ou plus au Questionnaire sur la santé du patient PHQ-9. Les registres sur la tuberculose ont fourni des données sur les défauts, l'échec ou la réussite des traitements ainsi que sur les décès. La qualité de vie a été évaluée au moyen d'une échelle visuelle analogique et nous avons calculé le score de handicap à l'aide de l'échelle d'évaluation du handicap (Disability Assessment Scale) de l'Organisation mondiale de la Santé. À l'aide d'une analyse de régression de Poisson multivariée, nous avons estimé l'association entre une dépression probable au début du traitement, les résultats du traitement et le décès.

**Résultats:**

Une dépression non traitée au début du traitement était associée de façon indépendante avec des défauts de traitement contre la tuberculose (risque relatif ajusté, RRa: 9,09; intervalle de confiance (IC) de 95%: 6,72 à 12,30), le décès (RRa: 2,99; IC 95%: 1,54 à 5,78), une augmentation du handicap (*β*: 0,83; IC 95%: 0,67 à 0,99) et une moins bonne qualité de vie (*β*: −0,07; IC 95%: −0,07 à −0,06) au bout de 6 mois. Les participants souffrant probablement d'une dépression avaient un score moyen de qualité de vie inférieur à ceux n'en souffrant pas (respectivement 5,0 contre 6,0; *P* < 0,001) et un score de handicap médian plus élevé (respectivement 22,0 contre 14,0; *P* < 0,001) au bout de 6 mois.

**Conclusion:**

La dépression non traitée chez les personnes atteintes de tuberculose était associée à de moins bons résultats du traitement, à une moins bonne qualité de vie et à un plus grand handicap. Les professionnels de santé devraient recevoir l'aide nécessaire pour proposer une prise en charge de la dépression aux personnes atteintes de tuberculose.

## Resumen

**Objetivo:**

Para investigar la asociación entre los resultados de la depresión concomitante y el tratamiento de la tuberculosis, la calidad de vida y la discapacidad en Etiopía.

**Métodos:**

El estudio incluyó 648 adultos consecutivos tratados contra la tuberculosis en 14 centros sanitarios primarios. Todos fueron evaluados en la iniciación del tratamiento (es decir, el principio) y tras 2 y 6 meses. Definimos la posible depresión como una puntuación de 10 o más en el Cuestionario de Salud del Paciente de nueve elementos. Los datos del defecto, incumplimiento y éxito del tratamiento y de la defunción se obtuvieron de los registros de la tuberculosis. La calidad de vida se evaluó usando una ampliación análoga visual y calculamos la puntuación de discapacidad usando la Ampliación de Asesoramiento de Discapacidad de la Organización Mundial de la Salud. Usando el análisis de regresión de Poisson multivariable, estimamos la asociación entre la posible depresión al principio y los resultados del tratamiento y la defunción.

**Resultados:**

La depresión sin tratar al principio estaba independientemente asociada con el defecto del tratamiento de la tuberculosis (coeficiente de riesgo ajustado, aRR: 9,09; intervalo de confianza, IC, del 95%: 6,72 a 12,30), defunción (aRR: 2,99; IC del 95%: 1,54 a 5,78), mayor discapacidad (*β*: 0,83; IC del 95%: 0,67 a 0,99) y calidad de vida más pobre (*β*: −0,07; IC del 95%: −0,07 a −0,06) en 6 meses. Los participantes con posible depresión tuvieron una puntuación de calidad de vida media más baja que aquellos sin (5,0 frente a 6,0, respectivamente; *P* < 0.001) y una puntuación de discapacidad media más alta (22,0 frente a 14,0, respectivamente; *P* < 0.001) en 6 meses.

**Conclusión:**

La depresión sin tratar en las personas con tuberculosis se asoció con los peores resultados del tratamiento, una calidad de vida más pobre y mayor discapacidad. Deberían darles el respaldo necesario a los trabajadores sanitarios para ofrecer atención a la depresión para personas con tuberculosis.

## ملخص

**الغرض:**

استقصاء العلاقة بين الاكتئاب المرضي ونتائج علاج السل، وجودة الحياة والعجز في إثيوبيا.

**الطريقة:**

تتضمن الدراسة 648 بالغًا على التوالي من الخاضعين لعلاج السل في 14 مرفقًا رئيسيًا لتوفير الرعاية الصحية. تم تقييم الجميع في بدء العلاج (أي خط الأساس) وبعد مرور شهرين و6 أشهر. وقد حددنا الاكتئاب المحتمل على مقياس من 10 أو أعلى على استبيان صحة المريض المكون من 9 عناصر. وتم الحصول على البيانات المتعلقة بنجاح العلاج أو فشله أو التخلف عنه من سجلات السل. وتم تقييم نوعية الحياة باستخدام مقياس النظير البصري وقمنا بحساب نتائج العجز باستخدام جدول تقييم العجز الخاص بمنظمة الصحة العالمية. وباستخدام تحليل انحدار بواسون متعدد المتغيرات، قمنا بتقييم العلاقة بين الاكتئاب المحتمل في خط الأساس ونتائج العلاج وحالات الوفاة.

**النتائج:**

ارتبط الاكتئاب غير المعالج في خط الأساس بشكلٍ مستقل بالتخلف عن علاج السل (نسبة الخطورة المصححة، aRR: 9.09؛ بنسبة أرجحية مقدارها ‏95‏‏%: 6.72 إلى 12.30)، حالات الوفاة (aRR: 2.99؛ ‏بنسبة أرجحية مقدارها 95‏%: 1.54 إلى 5.78)، العجز الأكبر (بيتا: 0.83؛ ‏بنسبة أرجحية مقدارها ‏95%: 0.67 إلى 0.99) ونوعية الحياة الأسوأ (بيتا: -0.07؛ ‏بنسبة أرجحية مقدارها 95‏%: -0.07 إلى -0.06) في 6 أشهر. يعاني المشاركون من أصحاب الاكتئاب المحتمل من متوسط نتيجة نوعية حياة أقل من هؤلاء الذين لا يعانون من الاكتئاب (5.0 في مقابل 6.0 على التوالي؛ نسبة الاحتمال <0.001) ونتيجة عجز وسطية أعلى (22.0 في مقابل 14.0 على التوالي؛ نسبة الاحتمال <0.001) في 6 أشهر.

**النتائج:**

يرتبط الاكتئاب غير المعالج لدى مرضى السل بنتائج علاج أسوأ، ونوعية حياة أسوأ، وعجز أكبر. ويجب منح العاملين في مجال الصحة الدعم اللازم لتوفير علاج الاكتئاب لمرضى السل.

## 摘要

**目的:**

调查埃塞俄比亚共病抑郁与肺结核治疗结果、生活质量和残疾程度之间的关系。

**方法:**

这项研究涉及 648 位连续在 14 个初级医疗机构接受肺结核治疗的成人。在治疗开始（即基准）和之后的 2 个月和 6 个月对所有患者进行了评估。我们在九项患者健康问卷中将潜在抑郁定义为 10 分或以上。从结核病登记处获得治疗常规、失败、成功和死亡数据。我们使用视觉模拟量表评估了生活质量，并使用世界卫生组织的残疾评估量表计算了残疾评分。我们使用多元 Poisson 回归分析，估计了基准处潜在抑郁、治疗结果和死亡之间的关联。

**结果:**

在 6 个月时，未治疗抑郁症的基准评估与肺结核治疗常规之间具有独立相关性（调整后的风险比，aRR: 9.09；95% 置信区间，CI: 6.72 至 12.30），死亡（aRR: 2.99；95% CI: 1.54 至 5.78），更高的残疾程度（*β*: 0.83；95% CI: 0.67 至 0.99）和更差的生活质量（*β*: ‑0.07；95% CI: ‑0.07 至 ‑0.06）。在 6 个月时，有潜在抑郁症的参与者的平均生活质量评分低于没有潜在抑郁症的参与者（分别为 5.0 和 6.0；*P* < 0.001），且其中位残疾评分更高（分别为 22.0 和 14.0；*P* < 0.001）。

**结论:**

结核病患者的未治疗抑郁症与较差的治疗效果、较低的生活质量和较高的残疾程度有关。应给予医疗工作者必要的支持，为结核病患者提供抑郁症护理。

## Резюме

**Цель:**

Изучить взаимосвязь между сопутствующей депрессией и результатами лечения туберкулеза, качеством жизни и инвалидизацией в Эфиопии.

**Методы:**

В исследовании участвовали 648 взрослых пациентов с туберкулезом, последовательно поступивших в 14 учреждений первичной медико-санитарной помощи. Все они прошли оценку в начале лечения (т. е. на исходном уровне), затем через 2 и 6 месяцев. Авторы считали депрессию вероятной, если пациент набирал 10 баллов и выше по шкале опросника о здоровье пациента, состоящей из 9 пунктов. Данные о прекращении лечения, отсутствии эффективности лечения, успешном лечении и летальных исходах были получены из журналов регистрации больных туберкулезом. Качество жизни оценивалось с использованием визуальной аналоговой шкалы, а степень инвалидизации была рассчитана по шкале оценки инвалидизации Всемирной организации здравоохранения. Используя многовариантный регрессионный анализ Пуассона, мы оценили взаимосвязь между вероятной депрессией на исходном уровне, результатами лечения и летальным исходом.

**Результаты:**

Наличие невылеченной депрессии на исходном уровне спустя 6 месяцев было независимо связано с прекращением лечения туберкулеза (скорректированный относительный риск, сОР: 9,09; 95%-й доверительный интервал, ДИ: от 6,72 до 12,30), с летальным исходом (сОР: 2,99; 95%-й ДИ: от 1,54 до 5,78), с более высокой степенью инвалидизации (*β*: 0,83; 95%-й ДИ: от 0,67 до 0,99) и с более низким качеством жизни (*β*: ‑0,07; 95%-й ДИ: от ‑0,07 до ‑0,06). Через 6 месяцев у пациентов с вероятной депрессией был более низкий средний показатель качества жизни, чем у пациентов без нее (5,0 против 6,0 соответственно, *P* < 0,001), и более высокий средний показатель степени инвалидизации (22,0 против 14,0 соответственно, *P* < 0,001).

**Вывод:**

Невылеченная депрессия у людей с туберкулезом была связана с худшими результатами лечения, более низким качеством жизни и большей степенью инвалидизации. Медицинским работникам должна быть предоставлена необходимая поддержка для оказания помощи при депрессии людям, больным туберкулезом.

## Introduction

Tuberculosis is the principal cause of death due to infectious disease worldwide;^1^ it accounts for 2.0% of the global disease burden, as measured in disability-adjusted life–years.^2^ In Ethiopia, tuberculosis is the fourth highest contributor to the disease burden.^3^ The World Health Organization’s (WHO’s) End-TB Strategy, launched in 2015, aims to achieve a treatment success rate of 90% by 2030 in all people with tuberculosis, including those with multidrug-resistant disease.^1^ People with tuberculosis often suffer from depression,^4^^–^^6^ which can reduce the likelihood of successful tuberculosis treatment,^7^ impair functioning^8^ and decrease quality of life.^9^ Systematic reviews have shown that depression is associated with poor medication adherence in people with human immunodeficiency virus (HIV) infections and acquired immune deficiency syndrome (AIDS).^10^ Moreover, in chronic noncommunicable diseases, depression has been observed to lead to poor treatment adherence and to lower immunity through neuroendocrine and behavioural mechanisms.^11^^,^^12^ These mechanism may also have a detrimental effect on responses to tuberculosis treatment.

Evidence on the impact of comorbid depression in tuberculosis is scarce.^7^ Although a few studies have assessed the association between depression and adherence to antituberculosis treatment, they were limited by small sample sizes of less than 70 patients. One study did analyse the relationship between depression and death or treatment discontinuation in people with tuberculosis,^13^ but we were unable to identify any study that disaggregated these outcomes. Other studies assessed disability^14^ and quality of life^15^ cross-sectionally or investigated changes from baseline in these variables after tuberculosis treatment.^8^ However, they did not evaluate the impact of comorbid depression, which is known to be an important cause of disability and poor quality of life in people with chronic disorders.^11^ Although global plans to end tuberculosis stress that both a patient-centred approach and social support are important for maximizing the treatment success rate, specific recommendations for people with comorbid depression is lacking.^1^

In addition, in low-income countries like Ethiopia, there are large gaps in treatment for mental health problems in general and for depression in particular.^16^ However, renewed efforts are being made to improve the detection and treatment of depression in primary health-care settings through WHO’s Mental Health Gap Action Programme (mhGAP).^17^ Greater understanding of the effect of untreated depression on the management of diseases important for public health, such as tuberculosis, is vital and would help ensure holistic care.

The aim of this study was to examine the impact of comorbid depression on treatment outcomes in people with tuberculosis in Ethiopia and on their health-related quality of life and level of disability.

## Methods

Between December 2014 and July 2016, we conducted a prospective observational study of people who were newly diagnosed with tuberculosis at 14 primary health-care centres in south central (i.e. in Silti and Gurage zones) and northern (i.e. Bahir Dar zone) Ethiopia. Two centres were hospitals and 12 were health centres. Facilities were eligible for inclusion if they had staff trained in mhGAP, including the detection and treatment of depression. We recruited study participants within 1 month of starting antituberculosis treatment and who: (i) were aged 18 years or older; (ii) had no plans to move out of the study area; (iii) were well enough to be interviewed, as judged by the interviewer or prospective participant; (iv) had not been an inpatient for more than 5 days in the previous month; and (vi) had not been diagnosed with multidrug-resistant tuberculosis. Between 23 December 2014 and 4 February 2015, we consecutively invited people who fulfilled these criteria to participate in the study by health professionals running tuberculosis clinics at the study centres. Trained nurse research assistants provided those willing to participate with detailed information and obtained written informed consent or witnessed a thumb print at data collection. In Ethiopia, all people with newly diagnosed tuberculosis are treated using the directly observed treatment, short course (DOTS) approach: a combination of rifampicin, ethambutol, isoniazid and pyrazinamide is administered for the first 2 months and, subsequently, a combination of rifampicin and isoniazid is given for an additional 4 months.^18^

### Study variables

The primary outcome variable was treatment default: a patient who defaulted was defined by the Ethiopian Federal Ministry of Health as one “who has been on treatment for at least four weeks and whose treatment was interrupted for eight or more consecutive weeks”.^18^ The timing of treatment default was taken to be midway between the last successful attempt to contact the person and the first unsuccessful attempt. Other treatment variables were treatment success, treatment failure and death due to any cause. Treatment was defined as successful if either the patient was cured (i.e. the sputum smear or culture became negative during, or in the last month of, treatment) or the treatment course was completed. Treatment was defined as having failed if the sputum smear or culture was positive 5 months or later after the start of treatment or a multidrug-resistant strain was present, irrespective of sputum smear or culture findings. Data on these variables were obtained from each centre’s tuberculosis register, which did not contain information after the time of referral on patients who were transferred to another area. The study protocol has been published elsewhere.^19^

The secondary outcome variables were quality of life and disability, which were assessed on three occasions over 6 months: (i) at baseline, at the start of the intensive treatment phase; (ii) at 2 months, after completion of the intensive treatment phase; and (iii) at 6 months, after completion of all tuberculosis treatment. Quality of life was assessed from responses to the question, “How would you rate your health-related quality of life?” and scored from zero for worst imaginable to 10 for best imaginable.^20^ Such single-item methods have been used successfully in population surveys, clinical settings and clinical interviews and found to be valid in indicating vulnerability to death due to all causes.^21^^,^^22^ No validated, tuberculosis-specific, quality-of-life instrument is available. Disability was assessed using the interviewer-administered version of the 12-item WHO Disability Assessment Schedule, version 2.0.^23^ This tool has been shown to be useful for assessing disability in primary care patients with depression and is able to capture changes over time.^24^^,^^25^ Moreover, it has been validated in Ethiopia and showed convergent validity with other predictors of impaired functioning in people with depression.^26^ At the three assessments, health professionals asked respondents if they were being treated for any mental illness, including depression.

Our exposure variable was probable depression which was identified using the nine-item version of the Patient Health Questionnaire and defined conservatively as a score of 10 or above.^27^^,^^28^ The nine-item version has been validated in two different treatment settings in Ethiopia.^28^^,^^29^ In the baseline assessment in our study, this version of the questionnaire was found to have construct validity and acceptable internal consistency, with an *α* of 0.81 and a mean inter-item correlation coefficient of 0.33.^6^ Participants who responded positively to the questionnaire item on suicidal ideation were referred for evaluation and treatment to health workers who had received training in mental health care as part of WHO’s mhGAP.^30^

We took into account a range of possible confounding variables such as age, sex, educational level, household income, marital status, religion, ethnicity and place of residence (i.e. urban versus rural). Data on these variables were obtained at baseline using a structured questionnaire. The duration of tuberculosis symptoms before diagnosis was self-reported by participants at baseline and information on the type of tuberculosis infection (i.e. pulmonary or extrapulmonary) was obtained from tuberculosis registers. The presence of any diagnosed comorbid chronic illnesses was also reported by participants themselves and whether or not they had an HIV infection, was recorded in tuberculosis registers. Use of substances, such as alcohol, tobacco and khat, was assessed using WHO’s Alcohol, Smoking and Substance Involvement Screening Test, version 3.1.^31^

Each participant’s perceived level of social support was assessed at baseline using the three-item Oslo-3 scale, which ranges from 3 to 14, with a high score indicating better perceived social support.^32^ This scale has previously been reported to work well in tuberculosis patients in Ethiopia.^33^ In addition, we assessed the stigma of tuberculosis at 2 months by adapting a 10-item tuberculosis stigma scale^34^ translated into Amharic;^19^ a high score indicated a high level of stigma. We also assessed participants’ perceptions about tuberculosis at 2 months: perceived tuberculosis severity was categorized as mild, moderate or severe; tuberculosis treatment was perceived as not helpful; somewhat helpful; or very helpful; and perceived barriers to tuberculosis treatment were identified from a yes or no answer to the question: “Are there barriers to taking your medications as prescribed?”

### Data analysis

Study variables and the participants’ characteristics are presented using descriptive statistics. We estimated the association between probable depression at baseline and treatment default, treatment success and death using multivariate Poisson regression analysis with a robust variance estimator and present the results as risk ratios.^35^ The follow-up time was included as a weighting variable in the analysis of these outcomes. We did not perform multivariate analysis for treatment failure because there were only six cases. We assessed differences in quality-of-life and disability scores between participants with and without probable depression at baseline and at 6 months using the independent samples *t* test and the Mann–Whitney *U* test. To examine the change in health-related quality-of-life and disability scores between tuberculosis diagnosis and the end of antituberculosis treatment, we used a multilevel, mixed-effects, generalized linear model to fit data from the three measurement times (i.e. baseline, 2 months and 6 months), with the three measurement times nested within individuals and individuals nested within each of the 14 primary care centres. The analysis was performed using Stata version 13.1 (StataCorp LP., College Station, United States of America) and study findings are reported in accordance with the Strengthening the Reporting of Observational Studies in Epidemiology (STROBE) statement.^36^

We calculated the study sample size using Stata version 12.0 for a power of 80%, a confidence level of 95% and an estimated prevalence of treatment default among tuberculosis patients without depression of 2.5%.^37^ In addition, the sample size had to be sufficient to detect a 5.0 percentage point increase in the prevalence of treatment default among people with comorbid depression when the ratio of nonexposure to exposure to depression was 2:1. With these parameters, the required sample size was 639, which was increased to 703 to include a 10% contingency for potential losses to follow up. The study was approved by the Institutional Review Board of the College of Health Sciences of Addis Ababa University (number 027/14/Psy).

## Results

**Fig. 1:**
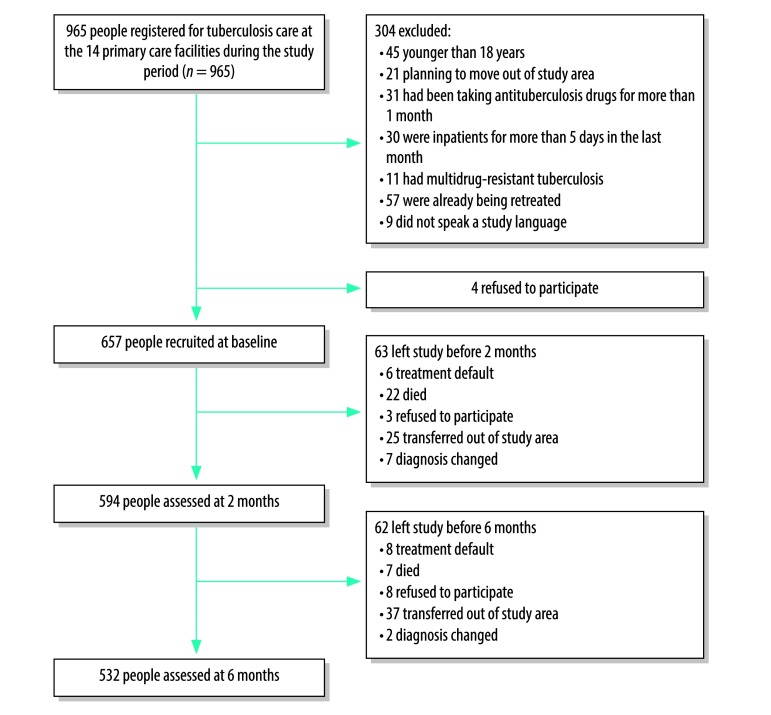
Flowchart, study of the effect of depression on tuberculosis treatment outcomes, Ethiopia, 2014–2016

In total, 657 people were recruited. However, as 9 people were subsequently found to have been misdiagnosed with tuberculosis, the study analysis included data on only 648. Participants’ ages ranged from 18 to 85 years and 53.7% (348/648) were male (Table 1). The median time between starting tuberculosis treatment and the first assessment was 0 days (interquartile range, IQR: 2). Face-to-face follow up assessments were conducted with 91.4% of those with tuberculosis (592/648) at 2 months and 82.1% (532/648) at 6 months. The median time from the start of antituberculosis treatment to the second assessment was 56 days (IQR: 1) and the median time to the third assessment was 160 days (IQR: 3). Data on treatment outcomes at 6 months were available for 88.7% (575/648) of participants. Overall, 9.6% (62/648) were transferred out of study sites (Fig. 1). At baseline, 53.9% (349/648) scored 10 or higher on the nine-item Patient Health Questionnaire and were classified as having probable depression (Table 2) here was no significant difference at baseline between those who completed the study and those who were transferred out in the frequency of probable depression, level of disability or quality of life.

**Table 1:** Sociodemographic characteristics of participants, study of the association between depression and tuberculosis treatment outcomes, Ethiopia, 2014–2016

Sociodemographic characteristic	No. of participants (%)^a^ *n* = 648
**Sex**	
Male	348 (53.7)
Female	300 (46.3)
**Age in years, mean (SD)**	30 (16.0)
**Marital status**	
Single	210 (32.4)
Married	358 (55.3)
Widowed or divorced	80 (12.4)
**Educational level**	
No formal education	224 (34.6)
Primary education	260 (40.1)
Secondary education or higher	164 (25.3)
**Occupation**	
Unemployed	37 (5.7)
Government employee	61 (9.4)
Self-employed	133 (20.5)
Farmer	172 (26.5)
Student	39 (6.0)
Homemaker	111 (17.1)
Day labourer	44 (6.8)
Other	51 (7.9)
**Annual household income in Ethiopian birr, mean (SD)^b^**	9 444 (13 200)
**Religion**	
Christian	429 (66.2)
Muslim	219 (33.8)
**Residence**	
Urban	364 (56.2)
Rural	284 (43.8)
**Ethnicity**	
Amhara	306 (47.2)
Gurage	192 (29.6)
Mareko	68 (10.5)
Silte	65 (10.0)
Other	17 (2.6)
**Perceived social support**	
Oslo-3 scale score, mean (SD)^c^	10 (4)
Tuberculosis stigma scale score, mean (SD)^d^	26 (10)

SD: standard deviation.

^a^ All values in the table represent absolute numbers and percentages unless otherwise stated.

^b^ In 2016, 1 Ethiopian birr = 22.5 United States dollars.

^c^ The Oslo-3 scale score indicates the participant’s perceived level of social support (range: 3 to 14), with a high score indicating better perceived social support.^32^

^d^ Tuberculosis stigma was assessed at 2 months in 592 participants using a 10-item scale (range: 10 to 50), on which a high score indicated a high level of stigma.

**Table 2:** Illness and substance use, study of the effect of depression on tuberculosis treatment outcomes, Ethiopia, 2014–2016

Variable	No. of participants (%) n = 648
**Probable depression^a^ at baseline**	349 (53.9)
**Suicidal ideation**	
No	535 (82.6)
Yes	113 (17.4)
**Duration of tuberculosis symptoms before diagnosis, weeks**	
< 2	40 (6.2)
2–12	338 (52.2)
13–52	209 (32.3)
> 52	61 (9.4)
**Type of tuberculosis**	
Pulmonary	371 (57.3)
Extrapulmonary	277 (42.8)
**HIV status**	
Negative	495 (76.4)
Positive	74 (11.4)
Unknown	79 (12.2)
**Hypertension**	1 (0.2)
**Heart disease**	3 (0.5)
**Diabetes mellitus**	5 (0.8)
**Previous depression**	0 (0.0)
**Alcohol use^b^**	
Low	562 (86.7)
Moderate	74 (11.4)
High	12 (1.9)
**Tobacco use^b^**	
Low	615 (94.9)
Moderate	29 (4.5)
High	4 (0.6)
**Khat use^b^**	
Low	544 (84.0)
Moderate	93 (14.3)
High	11 (1.7)
**Perceived tuberculosis severity^c^**	
Mild	62 (10.5)
Moderate	85 (14.4)
Severe	445 (75.2)
**Perceived benefit of tuberculosis treatment^c^**	
Not helpful	2 (0.3)
Somewhat helpful	23 (3.9)
Very helpful	567 (95.8)
**Perceived barriers to tuberculosis treatment^c^**	
No	458 (77.4)
Yes	134 (22.6)

HIV: human immunodeficiency virus.

^a^ Probable depression was defined as a score ≥ 10 on the nine-item version of the Patient Health Questionnaire.^27^

^b^ The use of substances, such as alcohol, tobacco and khat, was assessed at baseline using the World Health Organization’s Alcohol, Smoking and Substance Involvement Screening Test, version 3.1.^31^

^c^ Participants’ perceptions of tuberculosis severity, the benefit of tuberculosis treatment and barriers to tuberculosis treatment were assessed at 2 months in 592 participants.

At 6 months, the treatment default rate was significantly higher among participants with probable depression at baseline than among those without: 3.9% (12/309) versus 0.8% (2/266), respectively (*P* < 0.05). Similarly, the proportion who had died was significantly higher among those with probable depression: 7.8% (24/309) versus 1.9% (5/266) in those without (*P* < 0.01). In addition, the treatment success rate was significantly lower in those with probable depression: 87.1% (269/309) versus 96.6% (257/266) in those without (*P*  < 0.001; Table 3). On multivariate analysis, treatment default by 6 months was independently associated with probable depression (adjusted risk ratio, aRR: 9.09; 95% confidence interval, CI: 6.72 to 12.30), as was death (aRR: 2.99; 95% CI: 1.54 to 5.78). However, there was no significant association with treatment success (aRR: 0.95; 95% CI: 0.91 to 1.00), though the upper confidence bound was borderline for significance (Table 4).

**Table 3:** Tuberculosis treatment outcomes, by presence of probable depression, Ethiopia, 2014–2016

Indicator	Participants^a^		*P*
	With probable depression at baseline(*n* = 309)	Without probable depression at baseline(*n* = 266)	
**Treatment outcome, no. (%)**			
Treatment success	269 (87.1)	257 (96.6)	< 0.001
Treatment failure	4 (1.3)	2 (0.8)	ND
Treatment default	12 (3.9)	2 (0.8)	< 0.05
Death	24 (7.8)	5 (1.9)	< 0.01
**Quality-of-life score, mean (SD)^b,c^**			
At baseline before tuberculosis treatment	4.7 (2.7)	5.7 (2.4)	< 0.001
After 6 months of treatment	5.0 (2.4)	6.0 (2.2)	< 0.001
**Disability score, median(IQR)^c,d^**			
At baseline before tuberculosis treatment	30 (16)	18 (8)	< 0.001
After 6 months of treatment	22 (19)	14 (4)	< 0.001

IQR: interquartile range; ND: not determined; SD: standard deviation.

^a^ Treatment outcomes were known at 6 months for 575 of the 648 study participants: those who refused to be tested, were transferred to another area or whose diagnosis changed were excluded.

^b^ Quality of life was assessed using the question, “How would you rate your health-related quality of life?” and scored from 0 (worst) to 10 (best).

^c^ Quality-of-life and disability were assessed in 648 participants at baseline and in 532 at 6 months.

^d^ Disability was assessed using the interviewer-administered version of the 12-item World Health Organization Disability Assessment Schedule, version 2.0 (score range: 0 to 60).^23^

**Table 4:** Factors associated with tuberculosis treatment outcomes, Ethiopia, 2014–2016

Factor	Outcome							
	Treatment success			Treatment default			Death	
	cRR (95% CI)	aRR (95% CI)		cRR (95% CI)	aRR (95% CI)		cRR (95% CI)	aRR (95% CI)
**Probable depression^a^**								
No	Reference	Reference		Reference	Reference		Reference	Reference
Yes	0.96 (0.90 to 1.03)	0.95 (0.91 to 1.00)		5.53 (1.74 to 17.52)	9.09 (6.72 to 12.30)		4.42 (1.85 to 10.57)	2.99 (1.54 to 5.78)
**Sex**								
Male	Reference	Reference		Reference	Reference		Reference	Reference
Female	1.02 (1.02 to 1.03)	1.01 (1.01 to 1.01)		0.45 (0.31 to 0.63)	0.37 (0.33 to 0.43)		0.90 (0.44 to 1.88)	1.16 (0.56 to 2.41)
**Age, per year**	1.00 (1.00 to 1.00)	1.00 (1.00 to 1.00)		1.03 (1.01 to 1.05)	1.05 (0.98 to 1.12)		1.05 (1.04 to 1.05)	1.04 (1.02 to 1.07)
**Tuberculosis symptoms duration before diagnosis, weeks**								
≤ 12	Reference	Reference		Reference	Reference		Reference	Reference
> 12	0.99 (0.99 to 0.99)	0.99 (0.99 to 0.99)		0.57 (0.31 to 1.05)	0.44 (0.14 to 1.43)		1.51 (0.40 to 5.75)	1.57 (0.19 to 13.05)
**Household income, per 13 200-birr increase^b^**	0.99 (0.98 to 1.00)	0.99 (0.99 to 1.00)		0.81 (0.47 to 1.39)	1.28 (0.77 to 2.11)		0.85 (0.79 to 0.91)	0.95 (0.64 to 1.39)
**Residence**								
Urban	Reference	Reference		Reference	Reference		Reference	Reference
Rural	1.02 (1.01 to 1.04)	1.02 (1.02 to 1.03)		0.48 (0.29 to 0.81)	0.35 (0.25 to 0.49)		1.29 (1.13 to 1.48)	1.06 (0.96 to 1.17)
**Type of tuberculosis**								
Pulmonary	Reference	Reference		1.84 (1.49 to 2.27)	1.35 (0.76 to 2.40)		1.64 (1.15 to 2.34)	1.24 (0.39 to 3.89)
Extrapulmonary	1.01 (0.99 to 1.04)	1.00 (1.00 to 1.00)		Reference	Reference		Reference	Reference
**Educational level**								
No formal education	Reference	Reference		1.76 (0.64 to 4.82)	1.48 (0.16 to 13.54)		2.11 (0.55 to 8.11)	0.53 (0.03 to 10.93)
Primary education	1.0 (1.00 to 1.00)	1.00 (0.98 to 1.02)		2.18 (0.47 to 10.11)	2.05 (1.02 to 4.12)		2.02 (0.46 to 8.96)	0.95 (0.05 to 18.76)
Secondary education or higher	0.99 (0.96 to 1.03)	1.02 (0.98 to 1.05)		Reference	Reference		Reference	Reference
**Religion**								
Christian	Reference	Reference		Reference	Reference		Reference	Reference
Muslim	1.02 (1.00 to 1.04)	1.02 (0.97 to 1.06)		0.31 (0.25 to 0.38)	0.07 (0.05 to 0.11)		1.32 (0.55 to 3.16)	1.34 (0.93 to 1.92)
**Marital status**								
Single	Reference	Reference		Reference	Reference		Reference	Reference
Married	1.03 (1.02 to 1.04)	1.06 (1.03 to 1.09)		0.84 (0.20 to 3.49)	0.61 (0.18 to 2.11)		3.72 (1.16 to 11.96)	1.76 (0.12 to 25.89)
Widowed or divorced	1.03 (1.00 to 1.06)	1.07 (1.01 to 1.13)		2.47 (0.61 to 9.96)	1.71 (0.56 to 5.26)		4.94 (1.70 to 14.37)	1.73 (0.13 to 23.33)
**HIV status**								
Negative	Reference	Reference		Reference	Reference		Reference	Reference
Positive	0.96 (0.95 to 0.98)	0.97 (0.96 to 0.97)		ND^c^	ND^c^		1.55 (0.23 to 10.27)	2.04 (0.23 to 17.80)
Unknown	0.98 (0.94 to 1.02)	0.98 (0.93 to 1.03)		2.49 (0.80 to 7.77)	3.88 (0.79 to 18.99)		0.27 (0.21 to 0.34)	0.25 (0.19 to 0.32)
**Alcohol use^d^**								
Low	Reference	Reference		Reference	Reference		Reference	Reference
Moderate or high	0.95 (0.92 to 0.99)	0.96 (0.93 to 0.98)		1.84 (0.12 to 27.38)	0.48 (0.14 to 1.62)		0.78 (0.13 to 4.56)	0.90 (0.29 to 2.80)
**Khat use^d^**								
Low	Reference	Reference		Reference	Reference		Reference	Reference
Moderate or high	1.01 (0.98 to 1.04)	1.00 (0.98 to 1.02)		2.06 (0.70 to 6.07)	5.65 (2.18 to 14.63)		0.82 (2.61)	0.71 (0.09 to 5.68)
**Perceived social support**								
Oslo-3 scale, per 1-point increase^e^	1.00 (0.98 to 1.01)	1.00 (0.99 to 1.00)		0.81 (0.47 to 1.39)	0.91 (0.64 to 1.30)		1.10 (0.90 to 1.35)	1.13 (0.92 to 1.39)

aRR: adjusted risk ratio; CI: confidence interval; cRR: crude risk ratio; HIV: human immunodeficiency virus; ND: not determined.

^a^ Probable depression at baseline was defined as a score ≥ 10 on the nine-item version of the Patient Health Questionnaire.^27^

^b^ The standard deviation household income was 13200 Ethiopian birr (Table 2) and 1 Ethiopian birr = 22.5 United States dollars in 2016.

^c^ No participant with an HIV infection defaulted on treatment.

^d^ The use of substances, such as alcohol and khat, was assessed using the World Health Organization’s Alcohol, Smoking and Substance Involvement Screening Test, version 3.1.^31^

^e^ A high score on the Oslo-3 scale indicates better perceived social support.^32^

**Fig. 2:**
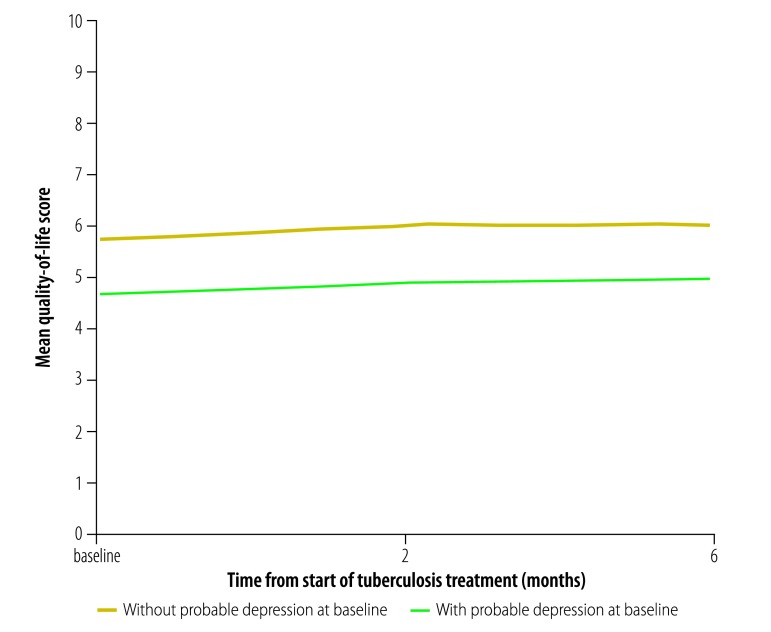
Change in quality of life with tuberculosis treatment, by depression at baseline, Ethiopia, 2014–2016

Note: Quality of life was assessed on the basis of responses to the question, “How would you rate your health-related quality of life?” and scored from 0 (worst) to 10 (best).

**Fig. 3:**
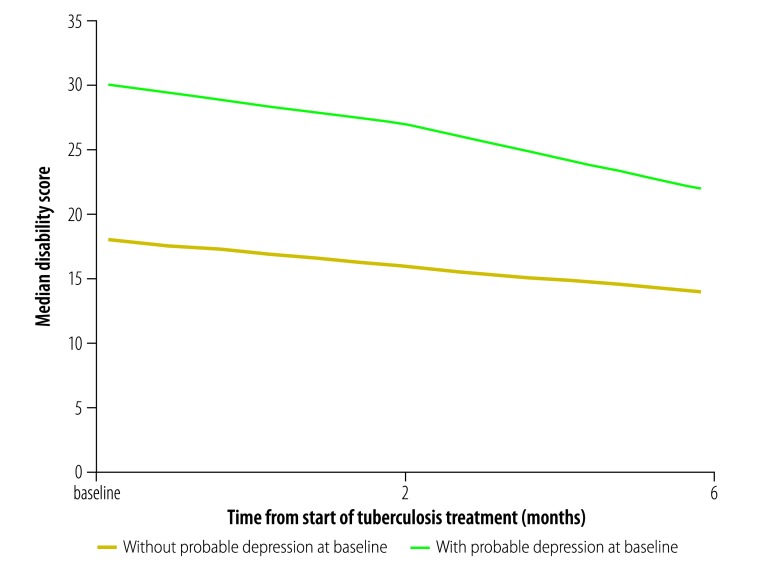
Change in disability score with tuberculosis treatment, by depression at baseline, Ethiopia, 2014–2016

Note: Disability was assessed using the interviewer-administered version of the 12-item World Health Organization Disability Assessment Schedule, version 2.0 (score range: 0–60).^23^

Probable depression at baseline was also associated with quality of life (Fig. 2) and disability (Fig. 3). The mean quality-of-life score at baseline was lower among those with probable depression than among those without (4.7 versus 5.7, respectively; *P* < 0.001) and the median disability score was higher (30.0 versus 18.0, respectively; *P* < 0.001). These differences remained significant at 6 months, when the mean quality-of-life score among those with and without probable depression was 5.0 and 6.0, respectively, (*P* < 0.001) and the median disability score was 22.0 and 14.0, respectively, (*P* < 0.001; Table 3). On multivariate analysis, quality of life at 6 months was significantly and negatively associated with probable depression (*β* = −0.07; 95% CI: −0.07 to −0.06) and disability was positively associated (*β* = 0.83; 95% CI: 0.67 to 0.99). There was no significant change over time in either mean quality-of-life score (*β* = −0.02; 95% CI: −0.13 to 0.09) or median disability score (*β* = −0.07; 95% CI: −0.33 to 0.22; Table 5). Data on factors other than depression that were associated with tuberculosis treatment outcomes are shown in Table 4 and data on factors associated with quality of life and disability are shown in Table 5.

**Table 5:** Factors associated with health-related quality of life and disability, study of the effect of depression on tuberculosis treatment outcomes, Ethiopia, 2014–2016

Factor	Quality-of-life score^a^			Disability score^b^	
	Crude *β* (95% CI)^c^	Adjusted *β* (95% CI)^c^		Crude *β* (95% CI)^c^	Adjusted *β* (95% CI)^c^
**PHQ-9 score,^d^ per 1-point increase**	−0.08 (−0.11 to −0.05)	−0.07 (−0.07 to −0.06)		0.92 (0.69 to 1.15)	0.83 (0.67 to 0.99)
**Time after start of tuberculosis treatment**	0.05 (−0.06 to 0.16)	−0.02 (−0.13 to 0.09)		−0.95 (−1.29 to −0.61)	−0.07 (−0.33 to 0.22)
**Sex**					
Male	Reference	Reference		Reference	Reference
Female	−0.14 (−0.44 to 0.15)	−0.04 (−0.18 to 0.11)		1.67 (0.48 to 2.86)	0.51 (0.09 to 0.93)
**Age**					
Below median	Reference	Reference		Reference	Reference
Median and above	−0.39 (−0.94 to 0.16)	−0.10 (−0.47 to 0.28)		3.37 (−0.42 to 7.16)	1.26 (−0.78 to 3.29)
**Tuberculosis symptoms duration before diagnosis, weeks**					
< 2	1.02 (0.31 to 1.72)	0.42 (−0.27 to 1.11)		−6.89 (−11.13 to −2.65)	−2.37 (−4.08 to −0.66)
2–12	0.22 (0.05 to 0.27)	−0.18 (−0.76 to 0.40)		−2.88 (−4.41 to −1.36)	−0.77 (−1.98 to 0.45)
13–52	0.01 (−0.24 to 0.27)	−0.42 (−0.98 to 0.15)		−1.79 (−2.82 to −0.75)	−0.43 (−0.78 to −0.07)
> 52	Reference	Reference		Reference	Reference
**Household income**					
Below median	Reference	Reference		Reference	Reference
Median and above	0.98 (0.75 to 1.21)	0.46 (0.45 to 0.47)		−1.39 (−2.67 to −0.11)	0.07 (−0.04 to 0.18)
**Residence**					
Urban	Reference	Reference		Reference	Reference
Rural	−0.47 (−1.00 to 0.07)	−0.35 (−0.47 to −0.22)		1.63 (1.10 to 2.15)	−0.01 (−1.20 to 1.19)
**Type of tuberculosis**					
Pulmonary	Reference	Reference		Reference	Reference
Extrapulmonary	0.14 (−0.25 to 0.53)	−0.05 (−0.13 to 0.03)		−1.76 (−2.41 to −1.11)	−0.92 (−1.38 to −0.46)
**Educational level**					
No formal education	−1.00 (−1.23 to −0.77)	−0.22 (−0.62 to 0.18)		5.66 (2.43 to 8.89)	1.47 (0.55 to 2.39)
Primary education	−0.51 (−0.55 to −0.47)	−0.12 (−0.24 to 0.00)		1.45 (−0.28 to 3.18)	−0.47 (−1.14 to 0.20)
Secondary education or higher	Reference	Reference		Reference	Reference
**Perceived tuberculosis severity**					
Mild	Reference	Reference		Reference	Reference
Moderate	−0.27 (−0.52 to −0.03)	−0.38 (−0.43 to −0.32)		−0.26 (−1.37 to 0.85)	0.22 (−0.80 to 1.23)
Severe	−0.74 (−1.10 to −0.37)	−0.45 (−0.58 to −0.33)		4.39 (3.23 to 5.56)	2.54 (1.37 to 3.71)
**Perceived barriers to tuberculosis treatment**					
No	Reference	Reference		Reference	Reference
Yes	−0.41 (−1.17 to 0.35)	−0.12 (−0.68 to 0.45)		3.24 (2.18 to 4.31)	1.44 (−0.05 to 2.93)
**Religion**					
Christian	Reference	Reference		Reference	Reference
Muslim	0.09 (−0.06 to 0.24)	0.11 (−0.12 to 0.34)		1.48 (0.09 to 2.87)	1.78 (1.66 to 1.90)
**Marital status**					
Single	Reference	Reference		Reference	Reference
Married	0.06 (−0.94 to 1.07)	0.40 (0.03 to 0.77)		2.20 (−3.02 to 7.41)	−0.51 (−1.67 to 0.64)
Widowed or divorced	−0.99 (−1.9 to −0.77)	−0.33 (−0.81 to −0.14)		5.42 (−1.56 to 12.39)	0.75 (−2.14 to 3.64)
**HIV status**					
Negative	Reference	Reference		Reference	Reference
Positive	−0.36 (−0.64 to −0.08)	−0.06 (−0.11 to −0.00)		1.82 (−0.54 to 4.17)	0.63 (−0.99 to 2.25)
Unknown	0.26 (0.23 to 0.28)	0.23 (0.09 to 0.38)		−0.63 (−1.40 to 0.30)	−0.13 (−0.29 to 0.04)
**Alcohol use^e^**					
Low	Reference	Reference		Reference	Reference
Moderate or high	−0.09 (−0.46 to 0.29)	0.13 (−0.55 to 0.82)		0.68 (−0.28 to 1.64)	0.96 (−0.06 to 1.97)
**Khat use^e^**					
Low	Reference	Reference		Reference	Reference
Moderate or high	−0.13 (−0.36 to 0.11)	−0.24 (−0.44 to −0.04)		−0.80 (−0.89 to −0.71)	−0.98 (−1.31 to −0.65)
**Perceived social support**					
Oslo-3 scale score, per 1-point increase^f^	0.27 (0.20 to 0.34)	0.20 (0.14 to 0.25)		−0.39 (−0.82 to 0.04)	0.05 (−0.36 to 0.47)
Tuberculosis stigma scale score, per 1-point increase^g^	−0.09 (−0.15 to −0.03)	−0.03(−0.08 to −0.01)		0.45 (0.39 to 0.51)	0.16 (0.15 to 0.16)

CI: confidence interval; HIV: human immunodeficiency virus; PHQ-9: nine-item version of the Patient Health Questionnaire.

^a^ Quality of life was assessed on the basis of responses to the question, “How would you rate your health-related quality of life?” and scored from 0 (worst) to 10 (best).

^b^ Disability was assessed using the interviewer-administered version of the 12-item World Health Organization Disability Assessment Schedule, version 2.0 (score range: 0 to 60).^23^

^c^ The *β* values were derived using a multilevel, mixed-effects, generalized linear model.

^d^ Probable depression at baseline was defined as a score ≥ 10 on PHQ-9, on which scores ranged from 0 to 27.^27^

^e^ The use of substances, such as alcohol and khat, was assessed using the World Health Organization’s Alcohol, Smoking and Substance Involvement Screening Test, version 3.1.^31^

^f^ A high score on the Oslo-3 scale indicates better perceived social support.^32^

^g^ A high score indicates greater stigma.

## Discussion

Our study provides evidence that people with tuberculosis in Ethiopia who had probable depression at the start of treatment were significantly more likely to default on treatment or die. Moreover, their chance of successful treatment was lower. Previous studies have also reported that depression compromises adherence to essential scheduled health care.^11^ With tuberculosis, treatment default leads to transmission of the infection to others, thereby raising the odds of further defaults,^38^ and increases the risk of multidrug-resistant disease.^39^

In agreement with our observations, systematic reviews and large population-based studies in both high- and low-income settings have found that mortality is increased in people with depression and that the association is maintained across patient groups.^40^^–^^43^ However, depression is a more serious concern for people with tuberculosis in Ethiopia because comorbid depression has been found in the majority.^6^ The mechanism by which depression increases mortality is likely to be complex. Although 113 of our 648 study participants reported suicidal ideation, we were not able to confirm its contribution to the mortality observed. One systematic review found that suicide contributed to less than 1.0% of deaths in medical samples like ours.^41^ Moreover, in our study area, the commonest cause of death in people with severe mental illness is infectious disease.^44^ In people with tuberculosis, depression may increase mortality through decreased self-care, including failure to take medications as prescribed,^11^ and through disability leading to poverty and substandard living conditions. One possible biological mechanism is depression-associated immune suppression.^45^

We also found that probable depression in people with tuberculosis was associated with poorer quality of life and greater disability, both at the start and after completion of antituberculosis treatment. In previous studies, neither quality of life nor the degree of disability returned to levels normal for the population by the end of tuberculosis treatment.^8^^,^^46^ Possible explanations are underlying depression, the quality of tuberculosis care falling short of international standards and the socioeconomic consequences of the illness and its associated stigma.^47^^,^^48^ One implication of these findings is that evaluating disability only during episodes of tuberculosis is likely to underestimate the disease burden as continuing disability after clinically successful treatment would be ignored.^49^

The treatment default rate we observed was markedly lower in females, rural residents and people living with HIV. Treatment adherence may have been stronger in people with an HIV infection, because of the counselling and additional support given to them in the Ethiopian health-care system, particularly in rural communities.^50^ Treatment default was also associated with higher khat use, substance use has previously been found to reduce the tuberculosis treatment success rate.^51^ In Ethiopia, the implementation of integrated care for people with mental, neurological and substance use disorders does not include khat use disorder as a target condition, because little is known about its adverse consequences and, because practical interventions are lacking. The level of disability was higher in females, in participants who perceived their episode of tuberculosis as severe, in those with pulmonary rather extrapulmonary tuberculosis and in those with a high level of stigma, indicating that both physical and psychosocial factors may contribute to the development of disability.^52^

Although we found that comorbid depressive symptoms in people with tuberculosis are often associated with poorer outcomes, even with successful treatment, generally health-care providers in Ethiopia do not assess depression in these people or provide evidence-based treatment. Consequently, unnoticed comorbid depressive symptoms may hamper efforts to end tuberculosis. National tuberculosis treatment guidelines may need to address depressive symptoms directly and health professionals should be trained to detect and treat depression in the context of the disease.

Our study has several of limitations. In our sample size calculation, we assumed that the tuberculosis treatment default rate in people without depression was 2.5%, which was based on a national report. We found a rate of 0.8%. However, we were still able to obtain estimates even with this sample size and do not believe it critically affected our findings. Second, in the low-income setting of our study, participants could have had undiagnosed, comorbid physical illnesses. Third, as we did not know whether or not participants were treated for depression outside the study centres, we may have overestimated the frequency of untreated depression. Nonetheless, as few people with depression receive treatment in Ethiopia, it is unlikely that misclassification of untreated depression seriously affected our results. Fourth, poverty may not have been fully captured by our sociodemographic variables and may have been a confounding factor. Fifth, as quality of life was assessed using a single question, no detailed information on different dimensions of quality of life was available. Sixth, we had no information on whether participants transferred out of the study area differed significantly from others in treatment outcomes or final quality-of-life or disability scores. However, there were no differences at baseline. Finally, our conclusions cannot be extended to tuberculosis patients who are hospitalized, are being retreated, or have multidrug-resistant disease. 

Nevertheless, consecutive patients were recruited and our study sample was reasonably representative, because several sites were included, all eligible people were invited to participate and data were collected over a long enough period to take seasonal variations into account.^53^ Use of the DOTS approach to tuberculosis treatment in Ethiopia means that our findings are generalizable to settings in low- and middle-income countries using a similar approach.

In conclusion, untreated depression appears to be a strong risk factor for treatment default and death in people with newly diagnosed tuberculosis and is associated with poor health-related quality of life and greater disability, despite successful tuberculosis treatment. Consequently, health-care workers should be given the support needed to provide depression care for people with tuberculosis.
